# Alterations in gene expression and sensitivity to genotoxic stress following HdmX or Hdm2 knockdown in human tumor cells harboring wild-type p53

**DOI:** 10.18632/aging.100008

**Published:** 2009-01-07

**Authors:** Katherine Heminger, Michael Markey, Meldrick Mpagi, Steven J. Berberich

**Affiliations:** Wright State University Boonshoft School of Medicine Biochemistry & Molecular Biology Department, Dayton, OH 45435, USA; ^1^current address: Procter and Gamble Co., Cincinnati OH 45241, USA

**Keywords:** p53, HdmX, Hdm2, RNAi, gene expression profiling

## Abstract

While half of all human tumors possess p53 mutations, inactivation of wild-type p53 can also occur through a variety of mechanisms that do not involve p53 gene mutation or deletion.  Our laboratory has been interested in tumor cells possessing wild-type p53 protein and elevated levels of HdmX and/or Hdm2, two critical negative regulators of p53 function. In this study we utilized RNAi to knockdown HdmX or Hdm2 in MCF7 human breast cancer cells, which harbor wild-type p53 and elevated levels of HdmX and Hdm2 then examined gene expression changes and effects on cell growth. Cell cycle and growth assays confirmed that the loss of either HdmX or Hdm2 led to a significant growth inhibition and G1 cell cycle arrest. Although the removal of overexpressed HdmX/2 appears limited to an anti-proliferative effect in MCF7 cells, the loss of HdmX and/or Hdm2 enhanced cytotoxicity in these same cells exposed to DNA damage. Through the use of Affymetrix GeneChips and subsequent RT-qPCR validations, we uncovered a subset of anti-proliferative p53 target genes activated upon HdmX/2 knockdown. Interestingly, a second set of genes, normally transactivated by E2F1 as cells transverse the G1-S phase boundary, were found repressed in a p21-dependent manner following HdmX/2 knockdown.  Taken together, these results provide novel insights into the reactivation of p53 in cells overexpressing HdmX and Hdm2.

## Introduction

Only half of all human tumors contain mutations in the p53 tumor suppressor gene [1], with the other half retaining wild-type p53 but possessing defects in the expression of p53 regulatory proteins and pathways.  Under non-stress conditions, p53 protein is maintained at a low basal level by constant ubiquitination and proteasomal degradation [2]. Upon DNA damage or various types of cellular stress, p53 is stabilized and functions as a transcription factor to induce genes involved in cell cycle arrest, apoptosis, and DNA repair [3].  The stringent regulation of p53 involves a complex network of proteins, and is critical for maintaining genomic stability and suppressing tumor formation.

Hdm2 and its structural homologue HdmX represent two essential negative regulators of p53 as demonstrated by their embryonic lethality in knockout mice and subsequent rescue by concurrent elimination of p53 [4].  Hdm2 inactivates p53 function through direct association resulting in an inhibition of transactivation [5] and, through its E3 ligase activity targeting p53, by ubiquitin-mediated proteasome degradation [6,7]. While HdmX shows conservation in the Hdm2 E3 ligase ring finger domain through which it can heterodimerize with Hdm2 [8,9], HdmX lacks the ability to ubiquitinate p53 in vivo [10,11] and thus can only antagonize p53 transactivation [12]. The heterodimerization of Hdm2 and HdmX also plays a critical role in the response to DNA damage enabling Hdm2 to promote the ubiquitination and rapid proteasomal degradation of HdmX, thereby facilitating the tumor suppressor activity of p53 [13-15].   Thus, the interactions between p53, Hdm2 and HdmX are critical for complete regulation of p53 [4].

The overexpression of either Hdm2 or HdmX can inhibit the activity of p53 and directly contribute to tumor formation.  It is not surprising that either one or both proteins are found overexpressed in many human tumors and tumor cell lines which harbor wild-type p53 [16].  Diverse approaches to activate the wild-type p53 in these tumors include the use of small molecule antagonists like Nutlin to inhibit the Hdm2-p53 interaction [17-19], and the use of antisense oligonucleotides, antibodies, and small interfering RNAs directed at Hdm2 or HdmX [20-23].  Recent findings suggest that Hdm2 and HdmX are specific independent therapeutic targets for activating wild-type p53 and that anti-cancer approaches that target both Hdm2 and HdmX should be considered as a means of treatment for tumors [16,18,24].

This study undertook an examination of gene expression alterations and the biological effects resulting from RNAi silencing of HdmX and Hdm2 in a breast cancer cell line overexpressing both proteins. Unlike previous studies examining only the biological effect of either HdmX or Hdm2 loss, this study focuses on a cell line where both proteins are overexpressed and further compliments those previous studies with a systematic examination of gene expression changes following loss of HdmX or Hdm2.  Interestingly, only p53 target genes primarily associated with cell cycle arrest were induced.  More striking was the repression of a large group of E2F-regulated genes upon HdmX/2 knockdown. Using siRNA approaches targeting p21, we were able to show that these E2F-regulated genes were repressed through p53 activation of p21.  Furthermore, cell proliferation and colony formation assays confirmed that loss of HdmX or Hdm2 inhibited tumor cell growth and could sensitize these cells to treatment with doxorubicin. Taken together, these results suggest that in cells where both Hdm2 and HdmX are overexpressed, removal of one leads to an anti-proliferative effect in tumor cells harboring wild-type p53 and induction of p53 cell cycle arrest genes that negatively feedback onto the E2F pathway.

## Results

### RNAi knockdown of Hdm2 and HdmX in MCF7 cells

**Figure 1.**
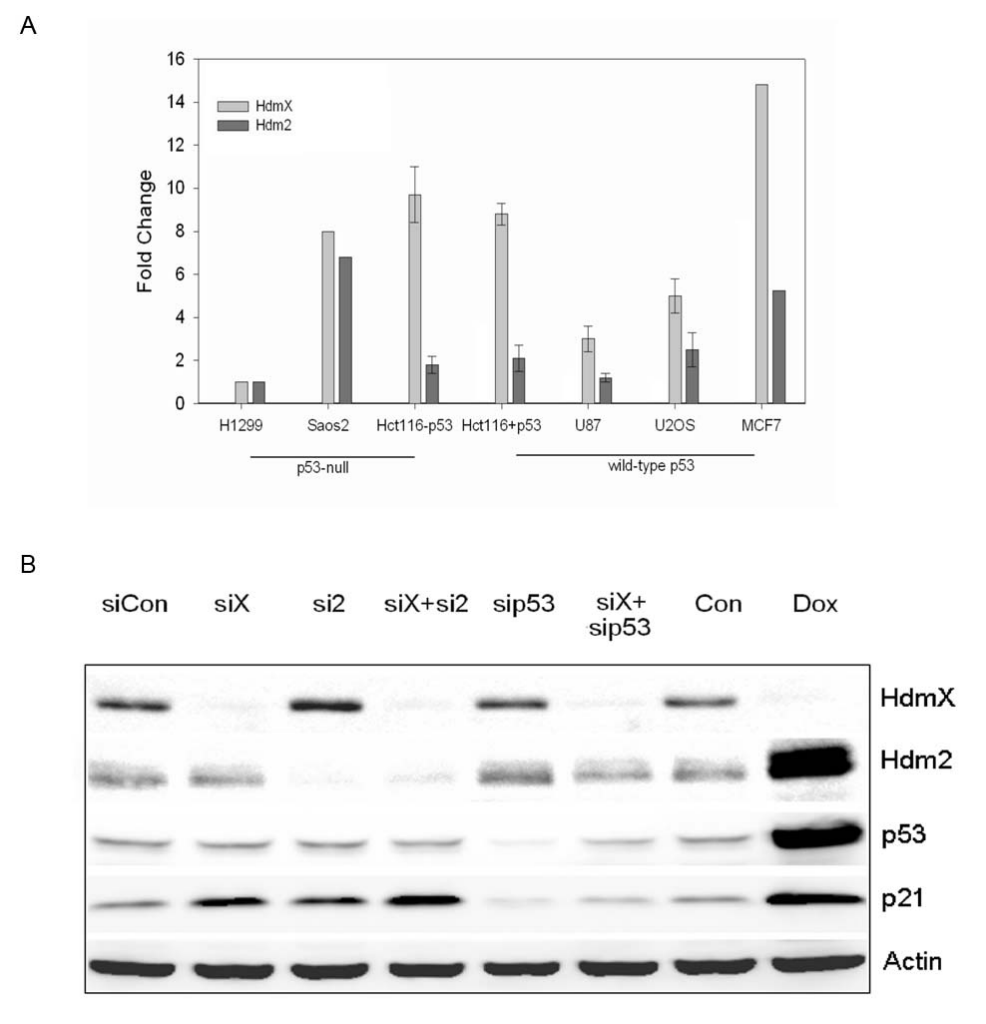
(**A**) RT-PCR analysis of *hdmX* and *hdm2*    gene expression in various human cell lines.  The endogenous levels of *hdmX*    and *hdm2* were determined relative to H1299 cells.  All samples were normalized to GAPDH. (**B**) RNAi knockdown of HdmX or Hdm2 triggers p53-dependent p21 induction. Western blot analysis of indicated proteins from the various siRNA or doxorubicin (Dox) treated MCF7 cells.  Knockdowns of the indicated proteins were greater than 80%. Protein extracts were made 24 hours after the last siRNA transfection or treatment with 5 μg/ml doxorubicin.

Given that HdmX and Hdm2 are overexpressed in approximately 17% of human tumors [16] the majority of which possess wild-type p53, this study set out to examine how loss of Hdm2/X affected gene expression and tumor cell growth.  MCF7, which possess wild-type p53 [25] and elevated levels of both HdmX and Hdm2 (Figure 1A) was the tumor cell line used in these studies. To inactivate HdmX and Hdm2 we employed siRNA targeting each gene as described in the materials and methods.

Before performing the Affymetrix GeneChip experiments we developed a triple transfection protocol that led to over 90% of the MCF7 cells taking up the siRNA (data not shown).            Next, the effectiveness of the knockdown was assessed using RT-qPCR (data not shown) and Western blotting. Following the triple transfection protocol HdmX and p53 protein levels were undetectable with Hdm2 showing a greater than 80% reduction in protein expression (Figure 1B). As expected, the loss of either HdmX or Hdm2 led to an increase in the levels of p21. This p21 increase is p53-dependent since no increase in p21 protein levels was detected upon concurrent knockdown of HdmX and p53.  While it has been suggested that Hdm2 controls the levels of p53 in non-stressed cells [26,27], in our hands MCF7 cells showed only a slight increase in p53 protein levels following the combined loss of HdmX and Hdm2. The inability of Hdm2 knockdown to result in an increase in p53 protein could be the result of MCF7 cells harboring an elevated level of HdmX.  Consistent with this suggestion, the treatment of MCF7 cells with Nutlin leads to increased p53 protein levels through loss of Hdm2 binding to p53 and concurrent Hdm2 mediated degradation of HdmX [28].

### Loss of Hdm2 and HdmX triggers inhibition of cell growth

**Figure 2.**
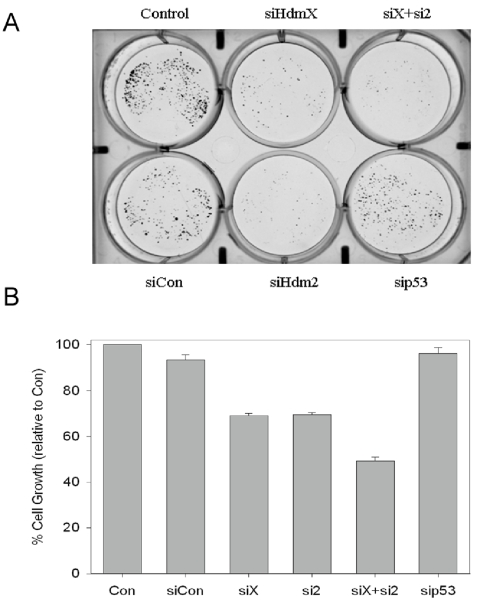
Loss of HdmX and/or Hdm2 inhibits MCF7 colony formation. (**A**) Following siRNA transfections, MCF7 cells were seeded at 500 cells/well in 6-well plates. The cells were allowed to grow for ten days then the colonies were stained with crystal violet. Significantly fewer colonies were present following knockdown of HdmX and/or Hdm2. The cells transfected with sip53 or a non-targeting control (siCon) showed minimal effects on colony formation relative to non-transfected control (Con/Control). (**B**) The percent cell growth relative to untransfected control was determined by extracting the stain in 10% acetic acid and quantifying the stain by reading absorbance at 590 nm.

Other groups have reported that in cells where wild-type p53 is kept in check by overexpression of HdmX or Hdm2, their inhibition can trigger alterations in cell growth [29] and in some conditions apoptosis [30]. To assess the growth properties of RNAi knockdown of p53 regulators Hdm2 and HdmX, siRNA-transfected MCF7 cells were plated at low density in 6 well plates and allowed to grow for an additional 10 days.  While transfection of siCon or sip53 resulted in only minimal changes in cell growth (Figure 2B), knockdown of  either HdmX or Hdm2, alone or in combination led to significantly fewer colonies (Figure 2A) and suppressed cell growth when compared to siCon (Figure 2B). This decrease in colony formation correlated with an increase in G1 arrest and not apoptosis (i.e. sub-G1) as determined by flow cytometry (data not shown).

### Loss of HdmX or Hdm2 sensitizes MCF7 cells to DNA damage

**Figure 3.**
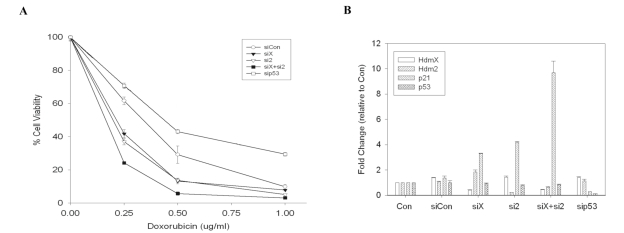
Knockdown of HdmX enhances doxorubicin-induced cytotoxicity. **(A)** Percent cell viability relative to untransfected untreated control cells. MCF7 cells were treated with doxorubicin (0.25-1.0 μg/mL) for 48 hours and cell viability was determined by absorbance at 590 nm. The loss of HdmX and/or Hdm2 showed an enhanced cytotoxicity relative to control cells. **(B)** RT-qPCR analysis of hdmX, hdm2, p21 and p53 gene expression in the indicated siRNA transfected MCF7 cells. The hdmX, hdm2, and p53 transcripts were effectively knocked down by siRNA prior to drug treatment.

Several recent studies using Nutlin and various DNA damaging agents reported that blocking Mdm2:p53 association led to increased chemosensitivity to DNA damaging agents [31,32]. To examine whether knockdown of HdmX and Hdm2 can also elicit increased cytotoxicity to DNA damage, MCF7 cells were transfected with the indicated siRNA leading to alterations of gene expression (Figure 3B). Cells were then treated with varying doses of doxorubicin and cell viability assessed.  siRNAs targeting HdmX or Hdm2 increased doxorubicin cytotoxicity, while removing both HdmX and Hdm2 led to the greatest level of chemosensitivity (Figure 3A).  Enhanced chemo-sensitivity was also observed in cisplatin treatment of siHdmX or siHdm2 MCF7 cells (data not shown).

### Gene expression profiles of MCF7 cells lacking HdmX or Hdm2

Having established an effective knockdown approach with effects on cell growth and increased sensitivity to DNA damage, we performed an Affymetrix GeneChip experiment to assess how loss of HdmX or Hdm2 affected global gene expression in MCF7 cells. Each RNAi transfection was performed in three separate bio-logical replicates.  The data analysis was carried out using GeneSpring GX software. Given the similarity of biological function uncovered in the previous experi-ments we focused our informatics on genes commonly altered following RNAi treatment with siHdmX or siHdm2. In summary, cel files were normalized using GCRMA, genes filtered by ANOVA and fold change, and genes significantly altered by both siHdmX and siHdm2 but not siHdmX + sip53 identified (see materials and methods for detailed approach).  From this approach we uncovered 394 gene alterations common to knockdown of both siHdmX and siHdm2 (Supplementary Table 1).

### p53 activation following loss of HdmX or Hdm2 triggers growth repressive genes

**Figure 4.**
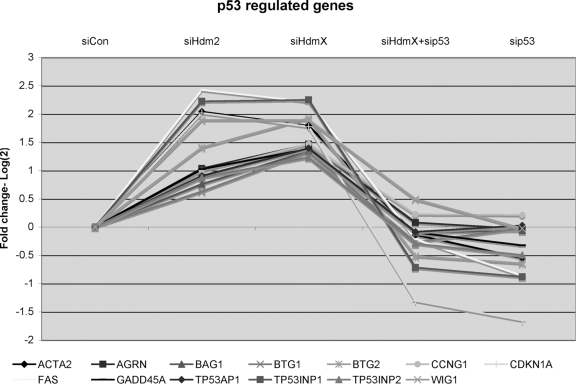
GeneChip expression of 13 known p53-regulated genes that were induced by knockdown of either siHdmX or siHdm2. Y-axis represents the average fold change (log_2_) for each of the genes in the indicated siRNA transfections relative to siCon (X-axis, conditions labeled at the top of the chart).

The initial examination of the 394 genes focused on those genes (n=222) that were increased following siHdmX or siHdm2 treatment relative to siCon.  Thirteen genes were identified that were known p53-regulated genes (Figure 4).  As expected these genes increased with siHdmX or siHdm2 treatment but had expression levels comparable or lower than siCon when treated with siHdmX+sip53 or sip53.  Interestingly, with the exception of Fas, this list of p53 target genes consisted predominately of genes encoding proteins involved in cell cycle arrest or DNA repair. Consistent with a model whereby p53 proapoptotic target genes require p53 that is phosphorylated at serine 46 by HIPK2 [33-35], we observed no detectable phosphorylation at serines 6, 15, 20, 46, or 392 following the RNAi transfection protocol employed in these studies (data not shown).

**Figure 5.**
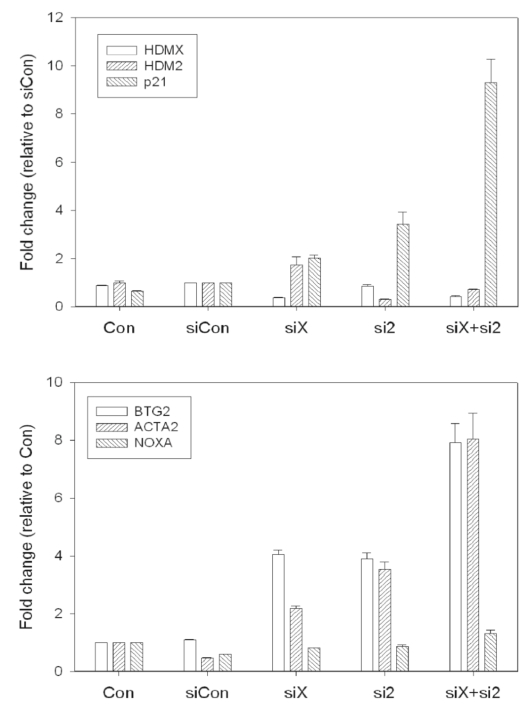
RT-qPCR validation of siRNA knockdown in MCF7 cells. (A). The hdmX, hdm2, and p21 mRNA expression relative to siCon (non-targeting siRNA) is shown. The p21 transcript is induced following loss of HdmX or Hdm2, and synergistically induced following loss of both HdmX and Hdm2. **(B)** BTG2, ACTA2, and NOXA mRNA expression relative to untransfected control (Con). The p53 target genes, BTG2 and ACTA2, are induced by loss of HdmX and/or Hdm2, while the expression of the proapoptotic gene, NOXA, is not altered.

To confirm these results, we performed RT-qPCR using TaqMan primers targeting five known p53 target genes, three of which were identified in our analysis.  p21, BTG2 and ACTA2 are p53 target genes that are associated with cell cycle arrest or growth inhibition [36-38], while Hdm2 is a negative regulator of p53 and Noxa a pro-apoptotic factor  not observed  in our list  of altered genes [39].  MCF7 cells were either mock transfected (Mock), transfected with siRNA that does not target any human gene (siCon) or transfected with siRNA to HdmX or Hdm2 either alone or in combination. The results in Figure 5 demonstrate that relative to siCon, knockdown of HdmX led to significant increases in hdm2, p21, BTG2 and ACTA2 gene expression.  No significant change in gene expression was observed with Noxa, which is consistent with our GeneChip results.  With the obvious exception of hdm2, siRNA-targeting Hdm2 led to similar alterations in gene expression (Figure 5).  Finally, when both HdmX and Hdm2 were eliminated, the levels of the cell cycle arrest genes p21, BTG2 and ACTA2 increased either synergistically or additively while levels of Noxa remained unchanged.  These results validate our GeneChip data that p53-target genes were induced upon HdmX or Hdm2 knockdown and that several of these genes encode proteins involved in the cell cycle arrest.

### p53 upregulation of p21 leads to global repression of E2F regulated genes

After searching for genes that were directly upregulated by p53 we next evaluated those genes that were repressed (N=172) following HdmX and Hdm2 knockdown (Figure 7).  Within the list of downregulated genes were a set of genes that encode proteins involved in G1-S phase transition, the majority of which were known E2F1 regulated genes.  It is known that p21 can inhibit CDK/cyclins involved in Rb phosphorylation [40] and within the literature we initially uncovered two reports where p53 activation led to repression of TERT or Chk2, two known E2F-regulated genes [41,42].  To determine whether repression of these genes was the result of an HdmX or Hdm2-dependent p53 activation, MCF7 cells were treated with siHdm2 or siHdmX alone or in combination with sip21.  RNA was isolated and RT-qPCR performed to monitor relative expression of cyclin A2 (CCNA2), p21 and E2F1.  While E2F1 did not make the 394 gene list, it possesses an E2F1 DNA binding site [43]. Relative expression for each of the genes was normalized to GAPDH.  As expected, loss of HdmX or Hdm2 led to an increase in p21 and concomitant decrease in both CCNA2 and E2F1 (Figure 7).  In contrast, loss of Hdm2/X and p21 completely abrogated CCNA2 and E2F1 repression consistent with p53 activation inactivating E2F1 transactivation via p21 induction.

## Discussion

As an essential tumor suppressor it is no surprise that human tumors demonstrate a diverse array of genetic mechanisms to inactivate p53 function.  Central to this present study are tumors where one or both of the negative regulators of p53, Hdm2 and HdmX, are overexpressed leading to loss of p53 activity.  Previous studies have focused on Hdm2 overexpression, where a small molecule inhibitor Nutlin 3 has proven to activate wild-type p53 in cell lines with elevated Hdm2, triggering apoptosis when combined with genotoxic agents that do not function as anti-mitotics [44]. Unfortunately, Nutlins have not proven as effective in tumors where HdmX is overexpressed [18,45-47], suggesting the need for additional approaches aimed at blocking the HdmX:p53 association particularly given the recent observation of HdmX overexpression in retinoblastoma [48].

Here we have employed RNAi approaches and DNA microarrays to better understand the activation of p53 in cells overexpressing Hdm2 and HdmX.  In MCF7 cells a growth arrest with no detectable apoptosis was observed following knockdown of either Hdm2 or HdmX (Figure 2 and data not shown).  While loss of either HdmX or Hdm2 was sufficient to trigger an anti-proliferative effect, the combined loss of both HdmX and Hdm2 resulted in a more significant growth inhibition.

**Figure 6.**
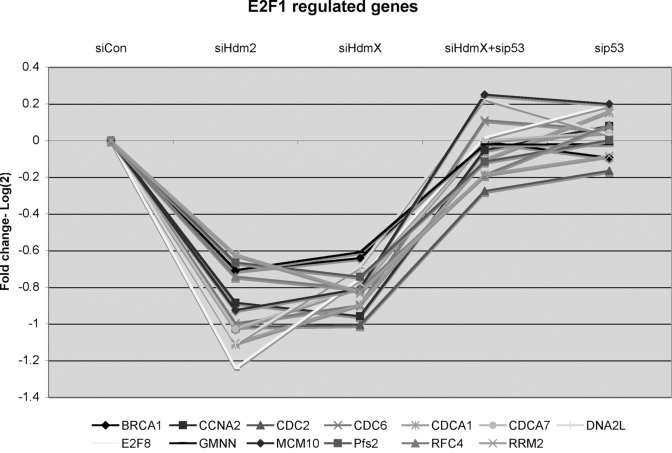
GeneChip expression of 13 reported E2F1-regulated genes that were repressed by knockdown of either siHdmX or siHdm2. Y-axis represents the average fold change (log_2_) for each of the genes in the indicated siRNA transfections relative to siCon (X-axis, conditions labeled at the top of the chart).

Even though this RNAi approach appears to activate p53 without triggering its phosphorylation (data not shown), the loss of either HdmX or Hdm2 did effectively sensitize the cells to doxorubicin with the loss of both Hdm2 and HdmX being most sensitive to DNA damage (Figure 3).  Surprisingly our results showed only a modest elevation of endogenous p53 levels following loss of HdmX and Hdm2 (Figure 1).  This result maybe unique to MCF7 cells which harbor elevated Hdm2 and HdmX, in contrast to most tumor cell lines with wild-type p53 that possessed only elevated Hdm2 (Figure 1A).   Consistent with the need for only one negative regulator to be elevated 65% of retinoblastoma tumors overexpress HdmX and possess wild-type p53 [48]. Based on our previous HdmX overexpression studies [10] we would predict that the overexpression of HdmX might inhibit Hdm2 degradation of p53 in MCF7 cells and thus could explain why modulating Hdm2 levels in MCF7 cells has no dramatic effect on p53 levels.

The DNA microarray experiment directly tested whether HdmX or Hdm2 knockdown triggered an increase in p53-regulated genes. While 394 genes were significantly altered by either HdmX or Hdm2 knockdown (Supplementary Table 1), only a small group was previously identified p53 targets (Figure 4).  A few of the remaining genes induced by HdmX or Hdm2 loss are likely novel p53 regulated genes (S. Berberich, personal communication) but most probably represent downstream effects of the cell cycle arrest induced by p53.  Within the 13 identified p53 target genes it is noteworthy that only one apoptotic gene (Fas) was found activated by loss of either HdmX or Hdm2. Upon careful examination of 16 known p53 pro-apoptotic genes we found that several of them were repressed following p53 knockdown, suggesting that their failure to be induced by loss of HdmX or Hdm2 was not a cell-type specific phenotype. Rather, we propose that the non-genotoxic release of p53 from Hdm2 of HdmX results in a preferential activation of growth arrest target genes, like p21 (Figure 5).  This model is consistent with recent work suggesting that p53 promoter selection is dependent on its phosphorylation [49].

**Figure 7.**
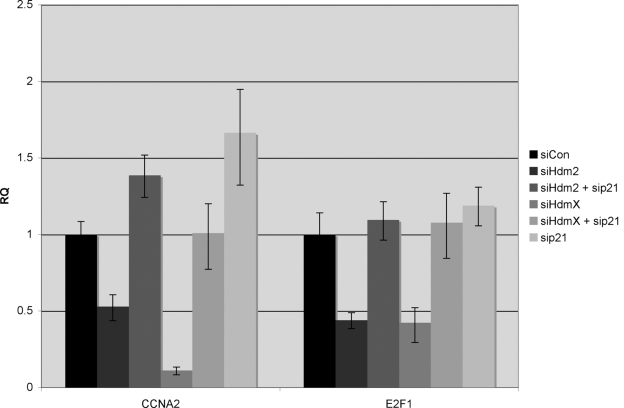
Repression of E2F1-regulated genes by Hdm2 or HdmX knockdown is blocked by concurrent knockdown of p21. MCF7 cells were transfected with the indicated siRNA combinations.  Twenty-four hours later, RNA was isolated and subjected to RT-qPCR to quantify expression of CCNA2, p21 and E2F1 after normalization to GAPDH.  Expression levels (Y-axis) were relative to siCon and reported as RQ values.  Error bars represent the 95% confidence interval of the relative expression.

Another interesting finding within the microarray data was a subgroup of genes that were repressed upon HdmX and Hdm2 knockdown and could be classified as known E2F-regulated genes. Other groups have noted that p53 activation of p21 could lead to the repression of TERT [42] or Chk2 [41], known E2F-target genes, and another group recently reported similar findings using microarray assays [50].

While this report focused on genes commonly regulated by HdmX and Hdm2, it is worth mentioning that within genes uniquely regulated by either HdmX or Hdm2 we did not observe any additional p53 regulated genes (M. Markey, personal communication). The common biological effects of HdmX or Hdm2-loss and significant overlap of gene expression patterns are in contrast to recent in vivo studies where the knockout of Mdm2 or MdmX in adult mouse tissues lead to non-overlapping roles in regards to regulating p53 activity [51].  We believe these findings point to either differences in cell culture verses tissue studies or more likely represent a significant departure in the roles that Hdm2 and HdmX play when expressed at physiological levels compared to the elevated levels in tumor cells.

Finally these studies demonstrate that non-genotoxic activation of p53 by knockdown of its inhibitors Hdm2 and HdmX leads to the induction of genes involved in cell-cycle arrest, as well as repression of genes along the E2F/Rb pathway that promote cell cycle entry. These alterations in gene expression resulted in a decreased population of proliferative cells without necessarily increasing apoptosis.  A non-genotoxic activation of p53 is one possible mechanism for the reduction in cellular proliferation observed during aging. This further underscores the critical importance of tumor suppressor activation in senescence and organismal aging.

## Materials and methods

Cell lines, antibodies, siRNA and chemotherapeutic agents. The human breast tumor cell line MCF7 was grown in Dulbecco's modified Eagle medium (DMEM) supplemented with 10% bovine growth serum (BGS), and 10 μg/ml gentamicin unless otherwise indicated.  HdmX polyclonal antibody (Bethyl Laboratories, Inc.), p21 polyclonal antibody C-19 (Santa Cruz Biotechnology, Inc.), p53 monoclonal antibody Ab-6 (Oncogene), Hdm2 monoclonal antibody SMP-14 (Santa Cruz Biotechnology, Inc.) and beta-actin monoclonal antibody (Sigma, Inc.) were used as indicated.  A phosphorylation-specific p53 polyclonal antibody kit (Cell Signaling Technology, Inc.) was utilized per manufacturer's protocol. Horseradish peroxidase (HRP)-conjugated anti-mouse or anti-rabbit secondary antibodies (Promega) were used with Super Signal substrate (Pierce) for chemiluminescence detection of proteins.    siGENOME duplex RNA targeting mRNA from hdmX, hdm2, or p53*,* and a non-targeting control siRNA were obtained from Dharmacon Research, Inc. and siRNA transfection was performed using Oligofectamine or Lipofectamine 2000 (Invitrogen) as described below. Doxorubicin hydro-chloride (Tocris Bioscience) was prepared as a 5 mg/ml stock solution in water.

siRNA transfection . Cells were seeded at 200,000 cells per well in 6-well plates (for RNA isolation), or at 700,000 cells per 6-cm dish (for protein extraction) in antibiotic free DMEM containing 1% BGS in a small volume.  Cells were reverse transfected with 100 nM siRNA (Dharmacon Research, Inc.) at time of seeding using Lipofectamine 2000 (Invitrogen).  After a five hour incubation, the media was removed and cells were refed with DMEM containing 10% BGS.  Twenty hours later, the cells were transfected again with 100 nM siRNA in a small volume of serum free media using Oligofectamine (Invitrogen).  After a four-hour incubation, an equal volume of DMEM containing 20% BGS was added to each well or dish without removing the transfection mixture.  Total RNA was isolated 24 hours post siRNA transfection and protein was extracted at 48 hours post siRNA unless otherwise indicated.

Analysis of Affymetrix GeneChips . The Affymetrix HG-U133 plus 2.0 GeneChips containing probe sets detecting over 54,000 transcripts were used in this study and each transfection condition was performed in triplicate. GeneChip cel files were imported into GeneSpring GX and preprocessed by GCRMA.  Measurements less than 0.01 were then set to 0.01, and each chip was normalized to the 50th percentile of the measurements taken from that chip.  Extra background correction was never applied.  Each gene was normalized to the median of the measurements for that gene, and then to the median of that gene's expression in the siCon condition.

Initially all genes were filtered in GeneSpring GX first by Welch ANOVA to find expression changes based on siRNA treatment, using a p-value cut off of 0.05 and the Benjamini and Hochberg False Discovery Rate as a multiple testing correction.  The cross-gene error model was active and based on replicates. From this list, genes were removed which varied between the mock and siCon treatments by 1.5 fold with a p-value < 0.05.  Next, lists of genes with expression changes of 1.5 fold and a p-value < 0.05 were then made for siHdm2 versus siCon and siHdmX versus siCon.  We then eliminated all but the union between these two lists.  One gene that was repressed in the siHdm2 condition but upregulated in the siHdmX condition (encoding hypothetical protein MGC5370) was manually removed.   Finally, genes that were not changed 1.5 fold with a p-value of <0.05 between the siHdmX and siHdmX + sip53 conditions were removed leaving a total of 394 selected genes.

Quantitative RT-pPCR . Cells were lysed directly in the culture dish and total RNA was isolated using the RNeasy kit (Qiagen) according to manufacturer's protocol.  The RNA was quantified by spectrophoto-meter reading at 260 nm, and 1 μg RNA was reverse transcribed with random hexamers to create cDNA using the TaqMan Reverse transcription kit (Applied Biosystems). Quantitative PCR was performed in a 96-well micro titer plate format on an ABI Prism 7900HT sequence detection system using 1 μl cDNA, TaqMan Universal PCR master mix and Assay-on-Demand Gene Expression products (Applied Biosystems) specific for genes of interest.  Each cDNA sample was analyzed in triplicate and fold change relative to control was calculated based on a PCR efficiency of two and normalized to GAPDH (endogenous control) RNA levels.  Average fold change and standard deviation were obtained from 2-3 biological replicate samples per treatment assayed in triplicate.

Western blot analysis . Frozen cells were lysed in an aqueous extraction buffer composed of 120 mM NaCl, 50 mM Tris-HCl (pH 8.0), 5 mM EGTA, 1 mM EDTA, 5 mM NaPPi, 10 mM NaF, 30 mM para-nitrophenylphosphate, 1 mM Benzamidine, 0.1% NP-40 (Ipegal Ca-630), 0.2 mM PMSF, and 1% protease inhibitor cocktail (Sigma), and soluble protein was recovered by centrifugation. Protein concentration was determined using Bradford reagent (Bio-Rad), and proteins were resolved on a sodium dodecyl sulfate-10% polyacrylamide gel followed by transfer of proteins to a polyvinylidene difluoride membrane (Millipore) using a Transblot system (Bio-Rad).  Immunoblotting was performed as previously described [52] using appropriate primary antibodies at 1:1000-1:10,000 dilution and secondary antibodies (goat anti-mouse or goat anti-rabbit HRP-conjugated, Promega) at 1:5000-1:10,000 dilution.  Blots were exposed to chemiluminescent reagent (Pierce) and protein was visualized on a FUJIFILM LAS-3000 image reader.

Colony formation and cell viability assays . Twenty-four hours after the second siRNA transfection, the cells were trypsinized, counted and seeded at 500 cells per well in 6-well plates for the colony formation assay.  The cells were allowed to grow for ten days, and then the colonies were fixed and stained in 1% crystal violet in 70% methanol.  The cell viability assays were performed in 96-well plates using either CellQuanti-Blue™ Reagent (BioAssay Systems) according to manufacturer's protocol or by staining the cells with crystal violet, extracting the stain in 10% acetic acid, and then reading absorbance at 590 nm.  Again, cells were trypsinized after the second siRNA transfection, counted and seeded at 20,000 cells per well.  Cell viability was determined at various time points post-seeding or following treatment with chemotherapeutic agents for the times indicated.

## Supplementary material

Supplementary Table 1 Genes deregulated by HdmX and Hdm2 in MCF7 cells.
