# Supplementary material for: Alterations in gene expression and sensitivity to genotoxic stress following HdmX or Hdm2 knockdown in human tumor cells harboring wild-type p53
Source: Aging (Albany NY). 2009 Jan 7;1(1):89–108. doi: 10.18632/aging.100008 (PMC2783638; doi:10.18632/aging.100008)
Supplement: Supplementary Table 1 [file aging-01-089-s001.doc]

**Supplementary Table 1. Genes deregulated by HdmX and Hdm2 in MCF7 cells.**

|  | **Fold Change vs. siCon** | | **Gene Symbol** |  |
| --- | --- | --- | --- | --- |
| **AffyID** | **siHdmX** | **siHdm2** |  | **Description** |
| 212354_at | 5.673 | 2.914 | SULF1 | sulfatase 1 |
| 205916_at | 5.6 | 2.427 | S100A7 | S100 calcium binding protein A7 (psoriasin 1) |
| 211163_s_at | 5.167 | 6.472 | TNFRSF10C | tumor necrosis factor receptor superfamily, member 10c, decoy without an intracellular domain |
| 206222_at | 4.986 | 7.782 | TNFRSF10C | tumor necrosis factor receptor superfamily, member 10c, decoy without an intracellular domain |
| 208180_s_at | 4.603 | 4.544 | HIST1H4H | histone 1, H4h |
| 206488_s_at | 4.424 | 3.301 | CD36 | CD36 antigen (collagen type I receptor, thrombospondin receptor) |
| 237737_at | 4.4 | 4.846 | LOC375010 ; LOC401131 | hypothetical LOC375010 ; hypothetical LOC401131 |
| 232035_at | 4.209 | 4.263 | HIST1H4H | histone 1, H4h |
| 216252_x_at | 3.97 | 5.55 | FAS | Fas (TNF receptor superfamily, member 6) |
| 213110_s_at | 3.929 | 3.082 | COL4A5 | collagen, type IV, alpha 5 (Alport syndrome) |
| 209555_s_at | 3.927 | 3.001 | CD36 | CD36 antigen (collagen type I receptor, thrombospondin receptor) |
| 229331_at | 3.756 | 4.49 | SPATA18 | spermatogenesis associated 18 homolog (rat) |
| 228766_at | 3.703 | 1.865 | CD36 | CD36 antigen (collagen type I receptor, thrombospondin receptor) |
| 208083_s_at | 3.664 | 3.871 | ITGB6 | integrin, beta 6 |
| 212097_at | 3.631 | 1.804 | CAV1 | caveolin 1, caveolae protein, 22kDa |
| 204781_s_at | 3.627 | 5.375 | FAS | Fas (TNF receptor superfamily, member 6) |
| 202917_s_at | 3.61 | 1.752 | S100A8 | S100 calcium binding protein A8 (calgranulin A) |
| 225912_at | 3.59 | 4.665 | TP53INP1 | tumor protein p53 inducible nuclear protein 1 |
| 215856_at | 3.493 | 2.626 | CD33L3 | CD33 antigen-like 3 |
| 215719_x_at | 3.479 | 5.572 | FAS | Fas (TNF receptor superfamily, member 6) |
| 226535_at | 3.47 | 3.972 | ITGB6 | integrin, beta 6 |
| 212344_at | 3.331 | 2.234 | SULF1 | sulfatase 1 |
| 202833_s_at | 3.198 | 1.717 | SERPINA1 | serpin peptidase inhibitor, clade A (alpha-1 antiproteinase, antitrypsin), member 1 |
| 209504_s_at | 3.138 | 3.235 | PLEKHB1 | pleckstrin homology domain containing, family B (evectins) member 1 |
| 218692_at | 3.104 | 2.476 | FLJ20366 | hypothetical protein FLJ20366 |
| 208096_s_at | 3.103 | 1.783 | COL21A1 | collagen, type XXI, alpha 1 ; collagen, type XXI, alpha 1 |
| 204780_s_at | 3.049 | 4.444 | FAS | Fas (TNF receptor superfamily, member 6) |
| 208683_at | 3.027 | 2.795 | CAPN2 | calpain 2, (m/II) large subunit |
| 219628_at | 2.982 | 3.714 | WIG1 | p53 target zinc finger protein |
| 211429_s_at | 2.976 | 1.724 | SERPINA1 | serpin peptidase inhibitor, clade A (alpha-1 antiproteinase, antitrypsin), member 1 |
| 1554062_at | 2.871 | 3.334 | XG | Xg blood group (pseudoautosomal boundary-divided on the X chromosome) |
| 207695_s_at | 2.847 | 1.969 | IGSF1 | immunoglobulin superfamily, member 1 |
| 212298_at | 2.819 | 2.164 | NRP1 | neuropilin 1 |
| 201236_s_at | 2.8 | 2.654 | BTG2 | BTG family, member 2 |
| 207392_x_at | 2.795 | 1.882 | UGT2B15 | UDP glucuronosyltransferase 2 family, polypeptide B15 |
| 215125_s_at | 2.784 | 1.964 | UGT1A10 ; UGT1A8 ; UGT1A7 ; UGT1A6 ; UGT1A5 ; UGT1A9 ; UGT1A4 ; UGT1A1 ; UGT1A3 | UDP glucuronosyltransferase 1 family, polypeptide A10 |
| 210387_at | 2.776 | 2.855 | HIST1H2BG | histone 1, H2bg |
| 208596_s_at | 2.739 | 2.215 | UGT1A10 ; UGT1A8 ; UGT1A7 ; UGT1A6 ; UGT1A5 ; UGT1A9 ; UGT1A4 ; UGT1A1 ; UGT1A3 | UDP glucuronosyltransferase 1 family, polypeptide A10 |
| 208084_at | 2.687 | 3.504 | ITGB6 | integrin, beta 6 |
| 242444_at | 2.665 | 2.379 | C1QTNF6 | C1q and tumor necrosis factor related protein 6 |
| 212998_x_at | 2.65 | 2.139 | HLA-DQB1 | major histocompatibility complex, class II, DQ beta 1 ; major histocompatibility complex, class II, DQ beta 1 |
| 202743_at | 2.648 | 2.41 | PIK3R3 | phosphoinositide-3-kinase, regulatory subunit 3 (p55, gamma) |
| 202688_at | 2.635 | 2.027 | TNFSF10 | tumor necrosis factor (ligand) superfamily, member 10 ; tumor necrosis factor (ligand) superfamily, member 10 |
| 205306_x_at | 2.633 | 3.434 | KMO | kynurenine 3-monooxygenase (kynurenine 3-hydroxylase) |
| 212347_x_at | 2.62 | 2.552 | MXD4 | MAX dimerization protein 4 |
| 211161_s_at | 2.581 | 1.685 | COL3A1 | collagen, type III, alpha 1 (Ehlers-Danlos syndrome type IV, autosomal dominant) |
| 227863_at | 2.577 | 2.524 | CTSD | cathepsin D (lysosomal aspartyl peptidase) |
| 220999_s_at | 2.573 | 3.036 | CYFIP2 | cytoplasmic FMR1 interacting protein 2 ; cytoplasmic FMR1 interacting protein 2 |
| 1559116_s_at | 2.559 | 1.995 | AD-020 | Chromosome 1 open reading frame 119 |
| 222150_s_at | 2.555 | 2.22 | LOC54103 | hypothetical protein LOC54103 |
| 206280_at | 2.533 | 1.924 | CDH18 | cadherin 18, type 2 |
| 228315_at | 2.528 | 3.249 |  | CDNA FLJ31683 fis, clone NT2RI2005353 |
| 1557779_at | 2.523 | 2.226 |  | Homo sapiens, clone IMAGE:4400004, mRNA |
| 200974_at | 2.52 | 4.108 | ACTA2 | actin, alpha 2, smooth muscle, aorta |
| 221756_at | 2.511 | 2.171 | MGC17330 | HGFL gene ; HGFL gene |
| 202180_s_at | 2.505 | 2.634 | MVP | major vault protein |
| 221218_s_at | 2.484 | 2.759 | TPK1 | thiamin pyrophosphokinase 1 |
| 219049_at | 2.479 | 1.767 | ChGn | chondroitin beta1,4 N-acetylgalactosaminyltransferase |
| 227020_at | 2.448 | 2.095 | YPEL2 | yippee-like 2 (Drosophila) |
| 225207_at | 2.441 | 2.274 | PDK4 | pyruvate dehydrogenase kinase, isoenzyme 4 |
| 215779_s_at | 2.439 | 2.315 | HIST1H2BG | histone 1, H2bg |
| 210778_s_at | 2.432 | 1.917 | MXD4 | MAX dimerization protein 4 |
| 202284_s_at | 2.428 | 4.05 | CDKN1A | cyclin-dependent kinase inhibitor 1A (p21, Cip1) |
| 211580_s_at | 2.405 | 1.801 | PIK3R3 | phosphoinositide-3-kinase, regulatory subunit 3 (p55, gamma) |
| 213261_at | 2.388 | 1.842 | LBA1 | lupus brain antigen 1 |
| 215785_s_at | 2.388 | 3.311 | CYFIP2 | cytoplasmic FMR1 interacting protein 2 |
| 210218_s_at | 2.381 | 2.055 | SP100 | nuclear antigen Sp100 |
| 215465_at | 2.375 | 2.541 | ABCA12 | ATP-binding cassette, sub-family A (ABC1), member 12 |
| 203058_s_at | 2.365 | 2.382 | PAPSS2 | 3'-phosphoadenosine 5'-phosphosulfate synthase 2 |
| 200984_s_at | 2.35 | 2.369 | CD59 | CD59 antigen p18-20 (antigen identified by monoclonal antibodies 16.3A5, EJ16, EJ30, EL32 and G344) |
| 225613_at | 2.346 | 2.243 | MAST4 | microtubule associated serine/threonine kinase family member 4 |
| 212463_at | 2.34 | 2.5 | CD59 | CD59 antigen p18-20 (antigen identified by monoclonal antibodies 16.3A5, EJ16, EJ30, EL32 and G344) |
| 204846_at | 2.338 | 2.398 | CP | ceruloplasmin (ferroxidase) |
| 236835_at | 2.336 | 1.973 | FUT8 | fucosyltransferase 8 (alpha (1,6) fucosyltransferase) |
| 236278_at | 2.333 | 2.228 |  |  |
| 214616_at | 2.322 | 2.008 | HIST1H3E | histone 1, H3e |
| 209737_at | 2.31 | 2.216 | MAGI2 | membrane associated guanylate kinase, WW and PDZ domain containing 2 |
| 203060_s_at | 2.304 | 2.3 | PAPSS2 | 3'-phosphoadenosine 5'-phosphosulfate synthase 2 |
| 1552632_a_at | 2.303 | 1.894 | KIAA1001 | Arylsulfatase G |
| 209460_at | 2.302 | 2.261 | ABAT | 4-aminobutyrate aminotransferase |
| 207664_at | 2.264 | 1.981 | ADAM2 | ADAM metallopeptidase domain 2 (fertilin beta) |
| 200696_s_at | 2.249 | 2.239 | GSN | gelsolin (amyloidosis, Finnish type) |
| 238439_at | 2.24 | 2.4 | ANKRD22 | ankyrin repeat domain 22 |
| 223315_at | 2.237 | 2.067 | NTN4 | netrin 4 |
| 224847_at | 2.237 | 2.597 | CDK6 | cyclin-dependent kinase 6 |
| 242093_at | 2.234 | 1.698 |  |  |
| 223686_at | 2.208 | 3.069 | TPK1 | thiamin pyrophosphokinase 1 |
| 210484_s_at | 2.204 | 3.38 | TNFRSF10C ; MGC31957 | tumor necrosis factor receptor superfamily, member 10c, decoy without an intracellular domain ; hypothetical protein MGC31957 |
| 201852_x_at | 2.203 | 1.924 | COL3A1 | collagen, type III, alpha 1 (Ehlers-Danlos syndrome type IV, autosomal dominant) |
| 1564573_at | 2.193 | 2.13 | LOC402778 | similar to RIKEN cDNA 6330512M04 gene (mouse) |
| 213744_at | 2.192 | 1.528 | ATRNL1 | attractin-like 1 |
| 229553_at | 2.192 | 1.863 | PGM2L1 | phosphoglucomutase 2-like 1 |
| 223600_s_at | 2.191 | 2.291 | KIAA1683 | KIAA1683 |
| 209160_at | 2.185 | 2.108 | AKR1C3 | aldo-keto reductase family 1, member C3 (3-alpha hydroxysteroid dehydrogenase, type II) |
| 211138_s_at | 2.18 | 2.362 | KMO | kynurenine 3-monooxygenase (kynurenine 3-hydroxylase) |
| 228390_at | 2.179 | 1.883 |  | CDNA clone IMAGE:5259272 |
| 206463_s_at | 2.172 | 2.849 | DHRS2 | dehydrogenase/reductase (SDR family) member 2 |
| 212346_s_at | 2.169 | 2.327 | MXD4 | MAX dimerization protein 4 |
| 1555756_a_at | 2.164 | 1.907 | CLEC7A | C-type lectin domain family 7, member A |
| 214455_at | 2.15 | 1.773 | HIST1H2BC | histone 1, H2bc |
| 228151_at | 2.148 | 1.989 |  | Transcribed locus |
| 1559322_at | 2.145 | 2.587 | PTP4A1 | Protein tyrosine phosphatase type IVA, member 1 |
| 203543_s_at | 2.127 | 1.708 | KLF9 | Kruppel-like factor 9 |
| 205776_at | 2.124 | 1.96 | FMO5 | flavin containing monooxygenase 5 |
| 206110_at | 2.122 | 2.096 | HIST1H3H | histone 1, H3h |
| 40016_g_at | 2.119 | 2.19 | MAST4 | microtubule associated serine/threonine kinase family member 4 |
| 205059_s_at | 2.114 | 2.441 | IDUA | iduronidase, alpha-L- |
| 202963_at | 2.113 | 2.189 | RFX5 | regulatory factor X, 5 (influences HLA class II expression) |
| 213664_at | 2.105 | 2.326 | SLC1A1 | solute carrier family 1 (neuronal/epithelial high affinity glutamate transporter, system Xag), member 1 |
| 218280_x_at | 2.101 | 2.187 | HIST2H2AA | histone 2, H2aa |
| 214696_at | 2.1 | 2.282 | MGC14376 | hypothetical protein MGC14376 |
| 225725_at | 2.091 | 2.788 |  | CDNA FLJ31683 fis, clone NT2RI2005353 |
| 224848_at | 2.075 | 2.117 | CDK6 | cyclin-dependent kinase 6 |
| 202964_s_at | 2.071 | 2.222 | RFX5 | regulatory factor X, 5 (influences HLA class II expression) |
| 238935_at | 2.07 | 2.278 | RPS27L | Ribosomal protein S27-like |
| 1568629_s_at | 2.055 | 2.066 | PIK3R2 | phosphoinositide-3-kinase, regulatory subunit 2 (p85 beta) |
| 223201_s_at | 2.055 | 1.808 | RP13-360B22.2 | hypothetical protein FLJ22679 |
| 218346_s_at | 2.037 | 2.688 | SESN1 | sestrin 1 |
| 202291_s_at | 2.026 | 1.539 | MGP | matrix Gla protein |
| 203887_s_at | 2.013 | 1.968 | THBD | thrombomodulin |
| 230093_at | 2.008 | 1.981 | TSGA2 | testis specific A2 homolog (mouse) |
| 219099_at | 2.002 | 2.401 | C12orf5 | chromosome 12 open reading frame 5 |
| 229441_at | 1.996 | 1.589 | PRSS23 | Protease, serine, 23 |
| 215076_s_at | 1.988 | 1.606 | COL3A1 | collagen, type III, alpha 1 (Ehlers-Danlos syndrome type IV, autosomal dominant) |
| 202073_at | 1.973 | 2.071 | OPTN | optineurin |
| 202357_s_at | 1.968 | 1.822 | BF | B-factor, properdin |
| 227221_at | 1.966 | 3 |  | CDNA FLJ31683 fis, clone NT2RI2005353 |
| 205110_s_at | 1.957 | 1.806 | FGF13 | fibroblast growth factor 13 |
| 203888_at | 1.953 | 1.979 | THBD | thrombomodulin |
| 203571_s_at | 1.941 | 1.905 | C10orf116 | chromosome 10 open reading frame 116 |
| 223878_at | 1.935 | 1.539 | INPP4B | inositol polyphosphate-4-phosphatase, type II, 105kDa |
| 223179_at | 1.934 | 2.243 | YPEL3 | yippee-like 3 (Drosophila) |
| 205174_s_at | 1.916 | 1.606 | QPCT | glutaminyl-peptide cyclotransferase (glutaminyl cyclase) |
| 218113_at | 1.908 | 1.569 | TMEM2 | transmembrane protein 2 |
| 235534_at | 1.908 | 2.274 |  | Homo sapiens, clone IMAGE:5723825, mRNA |
| 200983_x_at | 1.903 | 2.087 | CD59 | CD59 antigen p18-20 (antigen identified by monoclonal antibodies 16.3A5, EJ16, EJ30, EL32 and G344) |
| 211864_s_at | 1.885 | 2.053 | FER1L3 | fer-1-like 3, myoferlin (C. elegans) |
| 206482_at | 1.884 | 1.66 | PTK6 | PTK6 protein tyrosine kinase 6 |
| 223434_at | 1.883 | 1.588 | GBP3 | guanylate binding protein 3 |
| 223196_s_at | 1.877 | 2.476 | SESN2 | sestrin 2 |
| 1553033_at | 1.868 | 1.821 | SYTL5 | synaptotagmin-like 5 |
| 226771_at | 1.856 | 2.23 | ATP8B2 | ATPase, Class I, type 8B, member 2 |
| 201798_s_at | 1.855 | 1.889 | FER1L3 | fer-1-like 3, myoferlin (C. elegans) |
| 227134_at | 1.854 | 2.659 | SYTL1 | synaptotagmin-like 1 |
| 202708_s_at | 1.846 | 1.881 | HIST2H2BE | histone 2, H2be |
| 229566_at | 1.845 | 1.815 | LOC440449 | hypothetical gene supported by AF086204 |
| 205326_at | 1.823 | 1.651 | RAMP3 | receptor (calcitonin) activity modifying protein 3 |
| 238673_at | 1.82 | 1.691 |  | Transcribed locus |
| 222450_at | 1.818 | 1.724 | TMEPAI | transmembrane, prostate androgen induced RNA |
| 225927_at | 1.81 | 1.514 | MAP3K1 | mitogen-activated protein kinase kinase kinase 1 |
| 213142_x_at | 1.804 | 1.742 | LOC54103 | hypothetical protein LOC54103 |
| 1556308_at | 1.803 | 2.048 | FLJ33674 | hypothetical protein FLJ33674 |
| 225822_at | 1.803 | 2.181 | MGC17299 | hypothetical protein MGC17299 |
| 208796_s_at | 1.8 | 1.994 | CCNG1 | cyclin G1 |
| 226403_at | 1.795 | 1.785 | TMC4 | transmembrane channel-like 4 |
| 209333_at | 1.794 | 1.599 | ULK1 | unc-51-like kinase 1 (C. elegans) |
| 226864_at | 1.794 | 1.541 | PKIA | Protein kinase (cAMP-dependent, catalytic) inhibitor alpha |
| 203059_s_at | 1.787 | 2.03 | PAPSS2 | 3'-phosphoadenosine 5'-phosphosulfate synthase 2 |
| 214290_s_at | 1.773 | 1.898 | HIST2H2AA | histone 2, H2aa |
| 205726_at | 1.766 | 1.86 | DIAPH2 | diaphanous homolog 2 (Drosophila) |
| 219410_at | 1.753 | 1.508 | TMEM45A | transmembrane protein 45A |
| 37996_s_at | 1.752 | 1.871 | DMPK | dystrophia myotonica-protein kinase |
| 200766_at | 1.744 | 1.73 | CTSD | cathepsin D (lysosomal aspartyl peptidase) |
| 232306_at | 1.742 | 1.779 | CDH26 | cadherin-like 26 |
| 217419_x_at | 1.732 | 1.94 | AGRN | agrin |
| 219561_at | 1.723 | 1.849 | COPZ2 | coatomer protein complex, subunit zeta 2 |
| 216264_s_at | 1.718 | 1.998 | LAMB2 | laminin, beta 2 (laminin S) |
| 212120_at | 1.712 | 1.606 | RHOQ | Ras homolog gene family, member Q |
| 212285_s_at | 1.711 | 2.049 | AGRN | agrin |
| 218007_s_at | 1.707 | 1.645 | RPS27L | ribosomal protein S27-like |
| 230780_at | 1.707 | 1.809 |  | CDNA FLJ31839 fis, clone NT2RP7000086 |
| 207655_s_at | 1.7 | 2.051 | BLNK | B-cell linker |
| 231406_at | 1.7 | 1.665 | LOC401394 ; LOC402578 | hypothetical LOC401394 ; hypothetical LOC402578 |
| 204462_s_at | 1.699 | 1.527 | SLC16A2 | solute carrier family 16 (monocarboxylic acid transporters), member 2 |
| 214481_at | 1.699 | 1.542 | HIST1H2AM | Histone 1, H2am |
| 231766_s_at | 1.693 | 1.786 | COL12A1 | collagen, type XII, alpha 1 |
| 219687_at | 1.692 | 1.503 | HHAT | hedgehog acyltransferase |
| 202376_at | 1.69 | 1.527 | SERPINA3 | serpin peptidase inhibitor, clade A (alpha-1 antiproteinase, antitrypsin), member 3 |
| 204954_s_at | 1.689 | 2.736 | DYRK1B | dual-specificity tyrosine-(Y)-phosphorylation regulated kinase 1B |
| 208792_s_at | 1.677 | 1.825 | CLU | clusterin (complement lysis inhibitor, SP-40,40, sulfated glycoprotein 2, testosterone-repressed prostate message 2, apolipoprotein J) |
| 217529_at | 1.676 | 1.979 | LOC401394 ; LOC402578 | hypothetical LOC401394 ; hypothetical LOC402578 |
| 218471_s_at | 1.673 | 1.821 | BBS1 | Bardet-Biedl syndrome 1 |
| 203767_s_at | 1.664 | 1.758 | STS | steroid sulfatase (microsomal), arylsulfatase C, isozyme S |
| 208791_at | 1.663 | 2.024 | CLU | clusterin (complement lysis inhibitor, SP-40,40, sulfated glycoprotein 2, testosterone-repressed prostate message 2, apolipoprotein J) |
| 201648_at | 1.654 | 1.684 | JAK1 | Janus kinase 1 (a protein tyrosine kinase) |
| 209917_s_at | 1.646 | 1.874 | TP53AP1 | TP53 activated protein 1 |
| 212450_at | 1.645 | 1.534 | KIAA0256 | KIAA0256 gene product |
| 222043_at | 1.636 | 1.867 | CLU | clusterin (complement lysis inhibitor, SP-40,40, sulfated glycoprotein 2, testosterone-repressed prostate message 2, apolipoprotein J) |
| 204546_at | 1.625 | 2.033 | KIAA0513 | KIAA0513 |
| 236668_at | 1.621 | 1.853 |  | CDNA clone IMAGE:5312086 |
| 209623_at | 1.618 | 1.859 | MCCC2 | methylcrotonoyl-Coenzyme A carboxylase 2 (beta) |
| 209360_s_at | 1.616 | 1.583 | RUNX1 | runt-related transcription factor 1 (acute myeloid leukemia 1; aml1 oncogene) |
| 220613_s_at | 1.614 | 1.616 | SYTL2 | synaptotagmin-like 2 |
| 217767_at | 1.613 | 1.596 | C3 | complement component 3 |
| 209166_s_at | 1.611 | 1.58 | MAN2B1 | mannosidase, alpha, class 2B, member 1 |
| 207813_s_at | 1.61 | 3.021 | FDXR | ferredoxin reductase |
| 217783_s_at | 1.609 | 1.626 | YPEL5 | yippee-like 5 (Drosophila) |
| 201116_s_at | 1.608 | 1.764 | CPE | carboxypeptidase E |
| 209739_s_at | 1.6 | 1.817 | PNPLA4 | patatin-like phospholipase domain containing 4 |
| 219529_at | 1.59 | 1.596 | CLIC3 | chloride intracellular channel 3 |
| 223195_s_at | 1.59 | 2.022 | SESN2 | sestrin 2 |
| 203725_at | 1.589 | 2.034 | GADD45A | growth arrest and DNA-damage-inducible, alpha |
| 209216_at | 1.583 | 1.712 | WDR45 | WD repeat domain 45 |
| 234644_x_at | 1.582 | 1.738 |  | CDNA: FLJ22426 fis, clone HRC08780 |
| 214542_x_at | 1.581 | 1.637 | HIST1H2AI | histone 1, H2ai |
| 210886_x_at | 1.577 | 1.681 | TP53AP1 | TP53 activated protein 1 |
| 201939_at | 1.575 | 1.54 | PLK2 | polo-like kinase 2 (Drosophila) |
| 208890_s_at | 1.568 | 1.97 | PLXNB2 | plexin B2 |
| 211979_at | 1.561 | 1.549 | GPR107 | G protein-coupled receptor 107 |
| 210241_s_at | 1.557 | 1.585 | TP53AP1 | TP53 activated protein 1 |
| 210930_s_at | 1.557 | 1.629 | ERBB2 | v-erb-b2 erythroblastic leukemia viral oncogene homolog 2, neuro/glioblastoma derived oncogene homolog (avian) |
| 218706_s_at | 1.557 | 1.533 | NS3TP2 | HCV NS3-transactivated protein 2 |
| 202387_at | 1.545 | 1.691 | BAG1 | BCL2-associated athanogene ; BCL2-associated athanogene |
| 225968_at | 1.545 | 1.679 | PRICKLE2 | prickle-like 2 (Drosophila) |
| 200920_s_at | 1.54 | 1.545 | BTG1 | B-cell translocation gene 1, anti-proliferative |
| 216080_s_at | 1.539 | 1.537 | FADS3 | fatty acid desaturase 3 |
| 39248_at | 1.537 | 1.845 | AQP3 | aquaporin 3 |
| 217270_s_at | 1.535 | 1.844 | DYRK1B | dual-specificity tyrosine-(Y)-phosphorylation regulated kinase 1B |
| 214433_s_at | 1.534 | 1.634 | SELENBP1 | selenium binding protein 1 ; selenium binding protein 1 |
| 210224_at | 1.524 | 1.575 | MR1 | major histocompatibility complex, class I-related |
| 224836_at | 1.512 | 1.833 | TP53INP2 | tumor protein p53 inducible nuclear protein 2 |
| 212890_at | 1.511 | 1.874 | MGC15523 | hypothetical protein MGC15523 |
| 214086_s_at | 0.666 | 0.605 | PARP2 | poly (ADP-ribose) polymerase family, member 2 |
| 213346_at | 0.665 | 0.616 | LOC93081 | hypothetical protein BC015148 |
| 228559_at | 0.665 | 0.657 |  | CDNA clone IMAGE:6043059 |
| 227337_at | 0.663 | 0.544 | ANKRD37 | ankyrin repeat domain 37 |
| 235425_at | 0.663 | 0.554 | SGOL2 | shugoshin-like 2 (S. pombe) |
| 204435_at | 0.661 | 0.587 | NUPL1 | nucleoporin like 1 |
| 201890_at | 0.66 | 0.636 | RRM2 | ribonucleotide reductase M2 polypeptide |
| 220840_s_at | 0.66 | 0.588 | C1orf112 | chromosome 1 open reading frame 112 |
| 222843_at | 0.658 | 0.543 | FIGNL1 | fidgetin-like 1 |
| 204240_s_at | 0.657 | 0.631 | SMC2L1 | SMC2 structural maintenance of chromosomes 2-like 1 (yeast) |
| 228273_at | 0.657 | 0.612 | FLJ11029 | Hypothetical protein FLJ11029 |
| 203625_x_at | 0.656 | 0.579 | SKP2 | S-phase kinase-associated protein 2 (p45) |
| 218350_s_at | 0.656 | 0.613 | GMNN | geminin, DNA replication inhibitor |
| 219502_at | 0.656 | 0.492 | NEIL3 | nei endonuclease VIII-like 3 (E. coli) |
| 209608_s_at | 0.655 | 0.664 | ACAT2 | acetyl-Coenzyme A acetyltransferase 2 (acetoacetyl Coenzyme A thiolase) |
| 203213_at | 0.653 | 0.543 | CDC2 | Cell division cycle 2, G1 to S and G2 to M |
| 227787_s_at | 0.653 | 0.636 | THRAP6 | thyroid hormone receptor associated protein 6 |
| 219555_s_at | 0.65 | 0.621 | BM039 | uncharacterized bone marrow protein BM039 |
| 203302_at | 0.646 | 0.569 | DCK | deoxycytidine kinase |
| 222608_s_at | 0.646 | 0.54 | ANLN | anillin, actin binding protein (scraps homolog, Drosophila) |
| 222740_at | 0.646 | 0.473 | ATAD2 | ATPase family, AAA domain containing 2 |
| 216228_s_at | 0.645 | 0.554 | WDHD1 | WD repeat and HMG-box DNA binding protein 1 |
| 222848_at | 0.645 | 0.464 | FKSG14 | leucine zipper protein FKSG14 |
| 220865_s_at | 0.644 | 0.641 | TPRT | trans-prenyltransferase |
| 205394_at | 0.642 | 0.541 | CHEK1 | CHK1 checkpoint homolog (S. pombe) |
| 223256_at | 0.642 | 0.568 | KIAA1333 | KIAA1333 |
| 229442_at | 0.642 | 0.618 | C18orf54 | chromosome 18 open reading frame 54 |
| 204531_s_at | 0.641 | 0.61 | BRCA1 | breast cancer 1, early onset |
| 209754_s_at | 0.641 | 0.59 | TMPO | thymopoietin |
| 211767_at | 0.641 | 0.613 | SLD5 | SLD5 homolog ; SLD5 homolog |
| 223255_at | 0.641 | 0.554 | KIAA1333 | KIAA1333 |
| 225300_at | 0.641 | 0.639 | C15orf23 | chromosome 15 open reading frame 23 |
| 229886_at | 0.641 | 0.627 | FLJ32363 | FLJ32363 protein |
| 209709_s_at | 0.638 | 0.638 | HMMR | hyaluronan-mediated motility receptor (RHAMM) |
| 218755_at | 0.638 | 0.596 | KIF20A | kinesin family member 20A |
| 1568596_a_at | 0.637 | 0.657 | TROAP | trophinin associated protein (tastin) |
| 219531_at | 0.637 | 0.653 | Cep72 | centrosomal protein 72 kDa |
| 227545_at | 0.637 | 0.626 | BARD1 | BRCA1 associated RING domain 1 |
| 234944_s_at | 0.637 | 0.597 | FAM54A | family with sequence similarity 54, member A |
| 238075_at | 0.637 | 0.601 | CHEK1 | CHK1 checkpoint homolog (S. pombe) |
| 204962_s_at | 0.636 | 0.666 | CENPA | centromere protein A, 17kDa |
| 222039_at | 0.636 | 0.582 | LOC146909 | hypothetical protein LOC146909 |
| 202705_at | 0.635 | 0.651 | CCNB2 | cyclin B2 |
| 229610_at | 0.635 | 0.603 | FLJ40629 | hypothetical protein FLJ40629 |
| 219650_at | 0.634 | 0.605 | FLJ20105 | FLJ20105 protein |
| 201663_s_at | 0.633 | 0.618 | SMC4L1 | SMC4 structural maintenance of chromosomes 4-like 1 (yeast) |
| 218883_s_at | 0.633 | 0.641 | MLF1IP | MLF1 interacting protein |
| 209715_at | 0.632 | 0.621 | CBX5 | chromobox homolog 5 (HP1 alpha homolog, Drosophila) |
| 220239_at | 0.629 | 0.626 | KLHL7 | kelch-like 7 (Drosophila) |
| 209680_s_at | 0.628 | 0.531 | KIFC1 | kinesin family member C1 |
| 218768_at | 0.627 | 0.61 | NUP107 | nucleoporin 107kDa |
| 38158_at | 0.627 | 0.661 | ESPL1 | extra spindle poles like 1 (S. cerevisiae) |
| 204127_at | 0.626 | 0.6 | RFC3 | replication factor C (activator 1) 3, 38kDa |
| 209714_s_at | 0.625 | 0.629 | CDKN3 | cyclin-dependent kinase inhibitor 3 (CDK2-associated dual specificity phosphatase) |
| 235545_at | 0.625 | 0.556 | DEPDC1 | DEP domain containing 1 |
| 208955_at | 0.624 | 0.604 | DUT | dUTP pyrophosphatase |
| 201896_s_at | 0.623 | 0.65 | PSRC1 | proline/serine-rich coiled-coil 1 |
| 212621_at | 0.622 | 0.623 | KIAA0286 | KIAA0286 protein |
| 213647_at | 0.622 | 0.465 | DNA2L | DNA2 DNA replication helicase 2-like (yeast) |
| 204822_at | 0.62 | 0.523 | TTK | TTK protein kinase |
| 204825_at | 0.62 | 0.606 | MELK | maternal embryonic leucine zipper kinase |
| 215773_x_at | 0.62 | 0.664 | PARP2 | poly (ADP-ribose) polymerase family, member 2 |
| 204162_at | 0.619 | 0.573 | KNTC2 | kinetochore associated 2 |
| 205393_s_at | 0.619 | 0.561 | CHEK1 | CHK1 checkpoint homolog (S. pombe) |
| 221685_s_at | 0.619 | 0.555 | FLJ20364 | hypothetical protein FLJ20364 |
| 227928_at | 0.619 | 0.523 | FLJ20641 | hypothetical protein FLJ20641 |
| 228069_at | 0.619 | 0.575 | FAM54A | family with sequence similarity 54, member A |
| 230165_at | 0.619 | 0.548 | SGOL2 | shugoshin-like 2 (S. pombe) |
| 218585_s_at | 0.618 | 0.555 | DTL | denticleless homolog (Drosophila) |
| 218355_at | 0.616 | 0.638 | KIF4A | kinesin family member 4A |
| 223307_at | 0.616 | 0.63 | CDCA3 | cell division cycle associated 3 |
| 218039_at | 0.615 | 0.612 | NUSAP1 | nucleolar and spindle associated protein 1 |
| 204033_at | 0.614 | 0.628 | TRIP13 | thyroid hormone receptor interactor 13 |
| 225687_at | 0.613 | 0.621 | C20orf129 | chromosome 20 open reading frame 129 |
| 226308_at | 0.61 | 0.633 | NY-SAR-48 | sarcoma antigen NY-SAR-48 |
| 204752_x_at | 0.608 | 0.618 | PARP2 | poly (ADP-ribose) polymerase family, member 2 |
| 206653_at | 0.608 | 0.473 | POLR3G | Polymerase (RNA) III (DNA directed) polypeptide G (32kD) |
| 210983_s_at | 0.608 | 0.666 | MCM7 | MCM7 minichromosome maintenance deficient 7 (S. cerevisiae) |
| 218782_s_at | 0.608 | 0.5 | ATAD2 | ATPase family, AAA domain containing 2 |
| 219258_at | 0.608 | 0.527 | FLJ20516 | timeless-interacting protein |
| 208795_s_at | 0.607 | 0.634 | MCM7 | MCM7 minichromosome maintenance deficient 7 (S. cerevisiae) |
| 220060_s_at | 0.607 | 0.506 | FLJ20641 | hypothetical protein FLJ20641 |
| 221436_s_at | 0.607 | 0.623 | CDCA3 | cell division cycle associated 3 ; cell division cycle associated 3 |
| 223542_at | 0.607 | 0.512 | ANKRD32 | ankyrin repeat domain 32 |
| 1553244_at | 0.604 | 0.592 | FANCB | Fanconi anemia, complementation group B |
| 219004_s_at | 0.604 | 0.627 | C21orf45 | chromosome 21 open reading frame 45 |
| 221591_s_at | 0.603 | 0.647 | FAM64A | family with sequence similarity 64, member A |
| 203805_s_at | 0.602 | 0.59 | FANCA | Fanconi anemia, complementation group A ; Fanconi anemia, complementation group A |
| 219978_s_at | 0.601 | 0.579 | NUSAP1 | nucleolar and spindle associated protein 1 |
| 221879_at | 0.601 | 0.596 | CALML4 | calmodulin-like 4 |
| 203755_at | 0.6 | 0.577 | BUB1B | BUB1 budding uninhibited by benzimidazoles 1 homolog beta (yeast) |
| 203764_at | 0.6 | 0.567 | DLG7 | discs, large homolog 7 (Drosophila) |
| 204887_s_at | 0.6 | 0.59 | PLK4 | polo-like kinase 4 (Drosophila) |
| 206550_s_at | 0.6 | 0.61 | NUP155 | nucleoporin 155kDa |
| 227211_at | 0.6 | 0.577 | PHF19 | PHD finger protein 19 |
| 205053_at | 0.599 | 0.638 | PRIM1 | primase, polypeptide 1, 49kDa |
| 64408_s_at | 0.598 | 0.515 | CALML4 | calmodulin-like 4 |
| 221521_s_at | 0.597 | 0.63 | Pfs2 | DNA replication complex GINS protein PSF2 |
| 222962_s_at | 0.595 | 0.496 | MCM10 | MCM10 minichromosome maintenance deficient 10 (S. cerevisiae) |
| 205519_at | 0.594 | 0.533 | WDR76 | WD repeat domain 76 |
| 219990_at | 0.594 | 0.423 | E2F8 | E2F transcription factor 8 |
| 213226_at | 0.592 | 0.485 | CCNA2 | Cyclin A2 |
| 219703_at | 0.592 | 0.44 | MNS1 | meiosis-specific nuclear structural 1 |
| 242584_at | 0.589 | 0.516 | FLJ13305 | hypothetical protein FLJ13305 |
| 1552619_a_at | 0.587 | 0.48 | ANLN | anillin, actin binding protein (scraps homolog, Drosophila) |
| 204603_at | 0.583 | 0.503 | EXO1 | exonuclease 1 |
| 223570_at | 0.583 | 0.502 | MCM10 | MCM10 minichromosome maintenance deficient 10 (S. cerevisiae) |
| 204492_at | 0.582 | 0.628 | ARHGAP11A | Rho GTPase activating protein 11A |
| 214240_at | 0.582 | 0.643 | GAL | galanin |
| 219306_at | 0.582 | 0.56 | KIF15 | kinesin family member 15 |
| 203145_at | 0.581 | 0.645 | SPAG5 | sperm associated antigen 5 |
| 203968_s_at | 0.581 | 0.561 | CDC6 | CDC6 cell division cycle 6 homolog (S. cerevisiae) |
| 230847_at | 0.58 | 0.521 | WRNIP1 | Werner helicase interacting protein 1 |
| 221520_s_at | 0.578 | 0.662 | CDCA8 | cell division cycle associated 8 |
| 219294_at | 0.577 | 0.509 | C6orf139 | chromosome 6 open reading frame 139 |
| 1552921_a_at | 0.575 | 0.56 | FIGNL1 | fidgetin-like 1 |
| 224428_s_at | 0.575 | 0.491 | CDCA7 | cell division cycle associated 7 ; cell division cycle associated 7 |
| 218663_at | 0.573 | 0.579 | HCAP-G | chromosome condensation protein G |
| 1553984_s_at | 0.572 | 0.643 | DTYMK | deoxythymidylate kinase (thymidylate kinase) |
| 220651_s_at | 0.571 | 0.527 | MCM10 | MCM10 minichromosome maintenance deficient 10 (S. cerevisiae) |
| 236641_at | 0.571 | 0.48 | KIF14 | kinesin family member 14 |
| 204023_at | 0.57 | 0.598 | RFC4 | replication factor C (activator 1) 4, 37kDa |
| 205024_s_at | 0.568 | 0.584 | RAD51 | RAD51 homolog (RecA homolog, E. coli) (S. cerevisiae) |
| 218662_s_at | 0.566 | 0.516 | HCAP-G | chromosome condensation protein G |
| 222958_s_at | 0.566 | 0.473 | DEPDC1 | DEP domain containing 1 |
| 242787_at | 0.565 | 0.526 |  |  |
| 1554768_a_at | 0.564 | 0.522 | MAD2L1 | MAD2 mitotic arrest deficient-like 1 (yeast) |
| 204641_at | 0.564 | 0.521 | NEK2 | NIMA (never in mitosis gene a)-related kinase 2 |
| 209773_s_at | 0.564 | 0.65 | RRM2 | ribonucleotide reductase M2 polypeptide |
| 223229_at | 0.564 | 0.605 | UBE2T | ubiquitin-conjugating enzyme E2T (putative) |
| 201897_s_at | 0.563 | 0.602 | CKS1B | CDC28 protein kinase regulatory subunit 1B |
| 214804_at | 0.563 | 0.582 | FSHPRH1 | FSH primary response (LRPR1 homolog, rat) 1 |
| 225834_at | 0.562 | 0.489 | FAM72A | family with sequence similarity 72, member A |
| 202954_at | 0.56 | 0.641 | UBE2C | ubiquitin-conjugating enzyme E2C |
| 205909_at | 0.557 | 0.55 | POLE2 | polymerase (DNA directed), epsilon 2 (p59 subunit) |
| 205967_at | 0.553 | 0.556 | HIST1H4C | histone 1, H4c |
| 212949_at | 0.551 | 0.558 | BRRN1 | barren homolog (Drosophila) |
| 1553528_a_at | 0.548 | 0.438 | TAF5 | TAF5 RNA polymerase II, TATA box binding protein (TBP)-associated factor, 100kDa |
| 207891_s_at | 0.548 | 0.582 | TREX2 ; UIP1 | three prime repair exonuclease 2 ; 26S proteasome-associated UCH interacting protein 1 |
| 219494_at | 0.547 | 0.562 | RAD54B | RAD54 homolog B (S. cerevisiae) |
| 209891_at | 0.546 | 0.504 | SPBC25 | spindle pole body component 25 homolog (S. cerevisiae) |
| 205733_at | 0.545 | 0.521 | BLM | Bloom syndrome |
| 227165_at | 0.545 | 0.517 | C13orf3 | chromosome 13 open reading frame 3 |
| 210416_s_at | 0.544 | 0.572 | CHEK2 | CHK2 checkpoint homolog (S. pombe) |
| 215509_s_at | 0.544 | 0.493 | BUB1 | BUB1 budding uninhibited by benzimidazoles 1 homolog (yeast) |
| 37577_at | 0.544 | 0.538 | ARHGAP19 | Rho GTPase activating protein 19 |
| 212619_at | 0.542 | 0.501 | KIAA0286 | KIAA0286 protein |
| 211080_s_at | 0.541 | 0.524 | NEK2 | NIMA (never in mitosis gene a)-related kinase 2 ; NIMA (never in mitosis gene a)-related kinase 2 |
| 204128_s_at | 0.54 | 0.562 | RFC3 | replication factor C (activator 1) 3, 38kDa |
| 204126_s_at | 0.539 | 0.606 | CDC45L | CDC45 cell division cycle 45-like (S. cerevisiae) |
| 223381_at | 0.538 | 0.465 | CDCA1 | cell division cycle associated 1 |
| 203967_at | 0.536 | 0.501 | CDC6 | CDC6 cell division cycle 6 homolog (S. cerevisiae) |
| 220295_x_at | 0.535 | 0.444 | DEPDC1 | DEP domain containing 1 |
| 242939_at | 0.535 | 0.595 | TFDP1 | transcription factor Dp-1 |
| 222680_s_at | 0.533 | 0.532 | DTL | denticleless homolog (Drosophila) |
| 232278_s_at | 0.53 | 0.495 | DEPDC1 | DEP domain containing 1 |
| 204728_s_at | 0.529 | 0.501 | WDHD1 | WD repeat and HMG-box DNA binding protein 1 |
| 210053_at | 0.529 | 0.461 | TAF5 | TAF5 RNA polymerase II, TATA box binding protein (TBP)-associated factor, 100kDa |
| 206632_s_at | 0.522 | 0.526 | APOBEC3B | apolipoprotein B mRNA editing enzyme, catalytic polypeptide-like 3B |
| 202779_s_at | 0.517 | 0.642 | UBE2S | ubiquitin-conjugating enzyme E2S |
| 209464_at | 0.517 | 0.566 | AURKB | aurora kinase B |
| 203418_at | 0.514 | 0.541 | CCNA2 | cyclin A2 |
| 223700_at | 0.514 | 0.504 | GAJ | GAJ protein |
| 203214_x_at | 0.507 | 0.505 | CDC2 | cell division cycle 2, G1 to S and G2 to M |
| 218726_at | 0.507 | 0.527 | DKFZp762E1312 | hypothetical protein DKFZp762E1312 |
| 230021_at | 0.501 | 0.575 | MGC45866 | leucine-rich repeat kinase 1 |
| 209408_at | 0.498 | 0.564 | KIF2C | kinesin family member 2C |
| 211519_s_at | 0.498 | 0.537 | KIF2C | kinesin family member 2C |
| 210559_s_at | 0.497 | 0.494 | CDC2 | cell division cycle 2, G1 to S and G2 to M |
| 219000_s_at | 0.49 | 0.519 | DCC1 | defective in sister chromatid cohesion homolog 1 (S. cerevisiae) |
| 239002_at | 0.488 | 0.416 | ASPM | asp (abnormal spindle)-like, microcephaly associated (Drosophila) |
| 210334_x_at | 0.483 | 0.544 | BIRC5 | baculoviral IAP repeat-containing 5 (survivin) |
